# The Process of Disclosure: Mothers’ Experiences of Communicating X-Linked Carrier Risk Information to At-Risk Daughters

**DOI:** 10.1007/s10897-018-0251-7

**Published:** 2018-03-19

**Authors:** Amy Goldman, Alison Metcalfe, Rhona MacLeod

**Affiliations:** 1 Division of Evolution and Genomic Sciences, School of Biological Sciences, University of Manchester, Manchester, UK; 2 Manchester Centre for Genomic Medicine, St Mary’s Hospital, Manchester University NHS Foundation Trust, Manchester Academic Health Sciences Centre, 6th Floor, Oxford Road, Manchester, M13 9WL UK; 3 Faculty of Health and Well-being, Sheffield Hallam University, Sheffield, UK

**Keywords:** Family communication, X-linked, Carrier risk, Duchenne muscular dystrophy, Becker muscular dystrophy, Genetic counselling, Disclosure, Non-disclosure, Support, Carrier testing

## Abstract

When a boy is diagnosed with an X-linked condition such as Duchenne or Becker muscular dystrophy (D/BMD), the mother learns not only of her own potential carrier risk but also that of her daughters. Before the daughters are seen in the Genetics Clinic, responsibility for disclosing carrier risk information falls mainly to their mothers. We know little about if when and how these daughters are being told about their risk, and how mothers find the experience. Should we be doing more to help and support them? Using qualitative methods, six mothers known to the Manchester Centre for Genomic Medicine were interviewed about the disclosure of D/BMD carrier risk information to their daughters. The four key themes that arose are presented: communication process, facilitators of disclosure, barriers to disclosure and support and information. Despite the participants’ endeavours to be open and honest with their daughters and their belief that they had fully disclosed, key information was often withheld. Major barriers to discussion of the future, including reproductive options, were apparent. These were partly overcome by the involvement of genetic counsellors (GCs). The participants suggested a greater involvement of GCs, proactively sending appointments and written information, and offering carrier testing more flexibly.

**Electronic supplementary material:**

The online version of this article (10.1007/s10897-018-0251-7) contains supplementary material, which is available to authorized users.

## Introduction

Duchenne and Becker muscular dystrophies (DMD and BMD) are progressive muscle wasting disorders caused by mutations in the dystrophin gene and inherited in an X-linked manner, with symptoms usually apparent in early (DMD) or late (BMD) childhood (Emery 2002). Although a third of cases occur as de novo mutations in the affected male, the majority are inherited from a carrier mother (Haldane 1935). Therefore, a new diagnosis often coincides with the mother learning of her own potential carrier status and that of her daughters. Whilst female carriers are usually asymptomatic, 5–10% do manifest symptoms, which can include muscle weakness and cardiomyopathy (Grain et al. 2001; Politano et al. 1996; Van Westrum et al. 2011).

The decision about whether and when to undergo carrier testing can be complex and emotional, especially as the results can have major reproductive implications (Lewis et al. 2011). Kay and Kingston (2002) interviewed carriers of ‘serious’ X-linked conditions including nine DMD carriers and found that almost all intended to make reproductive decisions to avoid having an affected child. This highlights the importance of at-risk females knowing about their potential carrier status, genetic testing and reproductive options before starting a family, to enable them to make informed reproductive choices. However, due to the young onset of DMD and BMD, female siblings are often children themselves when the diagnosis in their family is made and their potential carrier risk is realised, so the initial responsibility for disclosing this information to the child falls mainly to the parents. Few studies have explored the communication of X-linked carrier risk information to at-risk daughters, so we know little about if when and how they are being told about their risk, and how families find the experience (Plumridge et al. 2010; Wilson et al. 2004).

In general, it is known that disclosure of genetic risk information to children is undertaken almost exclusively by mothers outside of the Genetics Clinic and tends to be a process rather than a single event (Forrest Keenan et al. 2009; McConkie-Rosell et al. 2009; Metcalfe et al. 2011, 2008; Rowland and Metcalfe 2013). Children who receive genetic risk information gradually and appropriately from a young age tend to find it less shocking and are able to cope and adapt to the information well by assimilating it into their self-identity as they grow up (Metcalfe et al. 2011, 2008; Plumridge et al. 2011). Following a meta-thematic synthesis of research into family communication of genetic risk information, Rowland and Metcalfe (2013) recommended that parents should disclose genetic risk information in an open and honest manner, at developmentally appropriate stages, gradually, throughout childhood and adolescence, whilst acknowledging and addressing their child’s emotions. Establishing an open and honest dialogue between the parent and child at an early stage makes it more likely the child will feel confident to approach their parent when questions arise, rather than them independently accessing potentially less accurate and worrying sources (Metcalfe et al. 2011, 2008; Plumridge et al. 2011).

Whilst effective disclosure of genetic risk information can lead to a stronger and more resilient family unit, limited disclosure can damage family relationships (Fanos and Puck 2001; Metcalfe et al. 2008, 2011; Plumridge et al. 2011). Plumridge et al. (2011) examined parental communication with siblings of children affected by one of six genetic conditions, inherited in various ways (including DMD), and found that conversations tended to focus around the health implications rather than the genetic risk. Disclosure of genetic risk information to children is a daunting task for many parents, who report feeling unsupported and ill-equipped (Metcalfe et al. 2008, 2011; Rowland and Metcalfe 2013). Parents often feel conflicted, weighing the need to provide valuable genetic information against the desire to protect their child from potential harm (Gaff et al. 2007). Stress, guilt and fear are prominent emotions and contribute to delayed, avoided or incomplete disclosure (Forrest Keenan et al. 2009; Metcalfe et al. 2011; Plumridge et al. 2011). Parents also deliberate over the optimal time for disclosure, concerned that they may harm their child by telling them information too early or too late, so they will often wait for their child to ask questions, which can result in delayed disclosure (Etchegary and Fowler 2008; Forrest Keenan et al. 2009; Metcalfe et al. 2008, 2011).

Plumridge et al. (2010) recognised that mothers from DMD families found talking to their children particularly difficult, often avoiding cues and leaving out important information, such as the inheritance pattern or the ‘Duchenne’ descriptor to prevent the children from finding out more information independently. As a result, the daughters in their cohort rarely understood about their carrier risk or testing options before age 16, which was later than for the other conditions they studied. They proposed that this might be due to the progressive and life-limiting nature of DMD being more difficult to discuss, or the high level of care required by the mothers being prioritised over providing information to their children. They also propose that the X-linked inheritance pattern leads to increased maternal guilt, impacting on their ability as the main communicator.

Although children and their parents tend to agree that the initial disclosure should be by the parents, who know the child best, they felt that this should be with the support and advice of health professionals (Forrest et al. 2008; McConkie-Rosell et al. 2009; Metcalfe et al. 2008; 2011; Plumridge et al. 2010, 2011). An international workshop on ‘Facilitating family adjustment to a diagnosis of Duchenne muscular dystrophy’ recommended that health care professionals should proactively encourage parents to discuss the condition with their children and ensure they are available to provide help and guidance for parents during the process (Poysky and Kinnett 2009). Rowland and Metcalfe (2013) concluded that a greater support is required from health professionals in order to facilitate family communication about genetic risk. They proposed using genetic counselling sessions to explore family communication patterns, suggest techniques and provide resources to assist parents in disclosing genetic risk information to their children.

In order to provide the required support, we must first understand more about this communication process. This study explores mothers’ experiences of communicating carrier risk information to daughters at risk of DMD or BMD.

## Methods

For this qualitative study, semi-structured telephone interviews were used to understand ‘What is the process of family^1^ communication of genetic carrier risk information from mothers to daughters (aged 12–18 years) who are potential carriers of DMD and BMD?’ The objectives were to explore:

1. The preparation for and communication of a DMD/BMD carrier risk to daughters aged 12–18 years
2. The perceived facilitators and barriers to the disclosure of this information
3. Support or information that was or would be helpful

### Participants

Purposive sampling was used to identify eligible participants: mothers with at least one daughter aged 12–18 years at the time of interview at risk of being a carrier, from a register of DMD and BMD families known to the Manchester Centre for Genomic Medicine. The mothers must have been aware of their own carrier status for at least 6 months prior to the interview to allow time for them to adjust to their own potential genetic status. Osborn and Smith (2008) recommend that five or six participants are a sufficient sample size—small enough to allow for a comprehensive analysis of each interview, but large enough to allow any similarities, differences, convergences and divergences to be examined. To account for the anticipated attrition, a total of 15 eligible participants were invited to participate.

### Procedures

An information pack was sent to the 15 eligible participants, inviting them to return a consent form if they wished to participate, with the choice of a face-to-face or telephone interview at a mutually convenient time. Interviews were semi-structured and comprised open-ended questions and prompts. The interviewer used a flexible topic guide (see Supplementary File) informed by the literature and developed with a research advisory group.^2^ Each interview was transcribed verbatim. The findings of the study were fed back to the participants as a summary leaflet.

### Data Analysis

An inductive thematic analysis was conducted to identify key themes that emerged around communication of genetic risk information from the interview data collected (Braun and Clarke 2006). From the transcripts, key quotes were highlighted and initial codes and observations noted down. Sub-themes that captured the meaning of a group of codes were ascribed. The researcher then analysed across the six data sets to cluster together related sub-themes. Each transcript was colour-coded to allow quotes, codes and sub-themes to be tracked back to the original data. Connections were made between the themes, allowing major themes to emerge (Miles and Huberman 1994).

## Results

Six participants took part (Table 1) and all requested telephone interviews. Four families were affected by DMD and two were affected by BMD. Five of the mothers were genetically confirmed carriers with an affected son aged between 10 and 23 years. Only one had a prior family history of the condition with a maternal uncle affected. One participant’s husband was affected by BMD, meaning her two daughters were obligate carriers. The participants had a total of nine daughters between them, aged 5–18 years old. Of these, four were untested and at 50% risk of being a carrier, two were obligate carriers, two had been tested (at age 4 and 15) and were carriers and one had been tested and was not a carrier (at age 14) (Table 1). The interviews were audio-recorded and lasted between 30 and 45 min. Of the nine non-participants, one declined to take part citing they were ‘too busy’ and eight did not respond.

**Table 1:** Participant details

Participant pseudonym	DMD/BMD family	Carrier mother?	Affected family member (age of son)	Age of daughter(s) (years)	Carrier risk? (age at carrier test)
Alicia	DMD	Yes	Son (21 years)	13	50%
Belinda	DMD	Yes	Son (10 years)	13 9	50% 50%
Claire	DMD	Yes	Son (10 years)	12 5	Carrier (4 years) 50%
Deborah	DMD	Yes	Son (20 years)	16	Non-carrier (14 years)
Eleanor	BMD	Yes	Son (23 years) and uncle	18	Carrier (15 years)
Fiona	BMD	No	Husband	18 14	Obligate carrier Obligate carrier

Four major themes arose: communication process, facilitators of disclosure, barriers to disclosure and support and information.

### Communication Process

The mothers felt that it was their responsibility to disclose carrier risk information to their daughters and had asserted the role of key information provider and gatekeeper of genetic information. They believed that it was better for them to be their daughters’ main source of information rather than their daughters seeking information from other sources, and felt that there was very little dialogue between their daughter and other family members, such as their father or siblings.

**Fig. 1:**
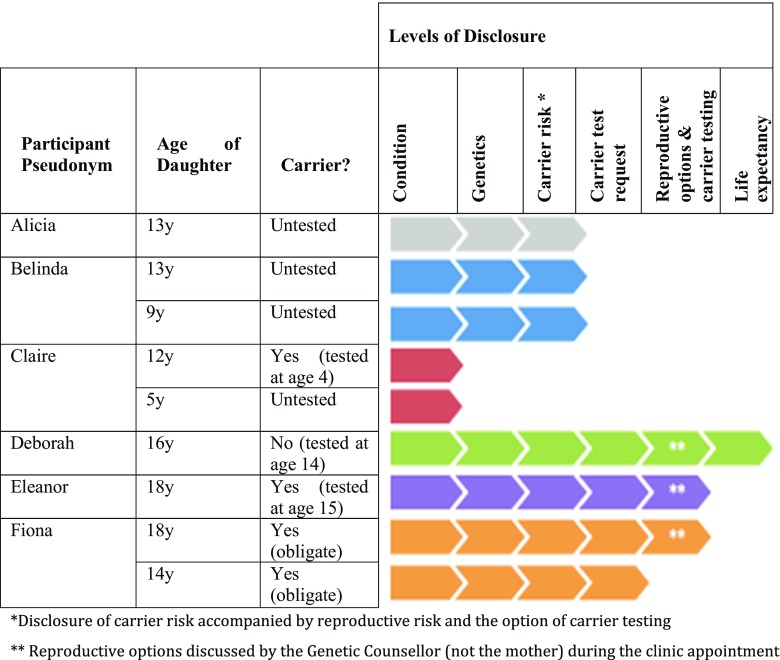
The six levels of carrier risk disclosure, demonstrating the partial disclosure at the time of interview (see Table 2 for descriptions of each level of disclosure)

It was evident from all the mothers that disclosure of carrier risk information was a process that took place over many years to allow their daughters to assimilate the information and prepare for the possibility of being a carrier. Six levels of disclosure were identified as follows (Fig. 1):

**Table 2:** Description of the six levels of carrier risk disclosure

Level of disclosure	Description
1. Condition	Initial discussions about D/BMD usually started from a young age and were often prompted by their daughters’ questions regarding their brother’s symptoms or hospital appointments. The mothers tended to use gentle language such as ‘poorly legs’ or ‘muscles not working properly’.
2. Genetics	The fact that D/BMD is an inherited genetic condition usually came a few years later. The mothers reported that they had instigated this conversation themselves and some had used diagrams to aid their explanations.
3. Carrier risk accompanied by reproductive risk and the option of carrier testing	Five of the six mothers stated they had disclosed their daughters’ carrier risk at an average age of 10 years old. They had used figures to represent risk, such as ‘it’s 50/50, we don’t know which’. During the same conversation, they informed their daughters that carrier testing would be available as a blood test when they were older. They had also disclosed the potential reproductive risk but had not gone into further detail about reproductive options.
4. Carrier test request	Deborah’s and Eleanor’s daughters subsequently requested carrier testing at age 12 and showed frustration at being told by their mothers that they could not be tested until they were age 16. Fiona’s daughters also requested genetic confirmation of their carrier status at around age 14.
5. Reproductive options and carrier testing	Deborah’s daughter, Eleanor’s daughter and Fiona’s eldest daughter were seen in the Genetics Clinic between ages 14 and 16, following their mothers’ request for an appointment. Their genetic counsellor discussed the reproductive options for the first time during their appointment, and carrier testing was performed in all three cases.
6. Life expectancy	Only one mother, Deborah, had discussed the reduced life expectancy with her son and daughter, as they had been aware of this since early childhood having been part of a family support group where many families had lost sons.

The majority of mothers had allowed their daughter to make an autonomous decision about carrier testing once they were aware of their risk. However, Claire had insisted that her eldest daughter was carrier-tested as an infant, soon after her son was diagnosed. Her daughter was tested and found to be a carrier at age 4. Interestingly, Claire had found disclosure of any carrier-related information significantly more difficult than the other mothers and had not informed either of her daughters about the genetics or inheritance of DMD/BMD:

*It’s something that I do need to discuss at some point in the near future, but I don’t know, there always seems to be a lot going on, and I haven’t got to that yet – Claire*

She had not disclosed her eldest daughter’s known positive carrier status to her at the time of interview (aged 12) and planned to disclose it ‘by the time she is in a serious relationship’. The planned timing of disclosures emerged as an important factor, with certain milestones repeatedly mentioned, for example when ‘moving to high school’, at ‘16 years old’ or when in ‘a serious relationship’. Sometimes these aided disclosure, but other times these delayed it:

*When she’s 16 if she wants to have blood tests then, you know, I will go into a bit more depth with her then. I mean I feel like I could talk to her now about it but I don’t see much point – Alicia*

However, for those who had disclosed more information, the timings had often been unplanned, with conversations prompted by their daughters’ questions or sons’ hospital appointments.

### Facilitators of Disclosure

Many participants cited their perceived ‘open and honest’ communication style in addition to remaining ‘positive’ as facilitators of the disclosure process. They felt that this was the optimum style of communication, and that if done from a young age, it would prevent a possible adverse reaction upon discovery of their risk information:

*I’ve been honest with them from the word go. I won’t keep things from them because I don’t want them getting to a certain age and then saying, ‘What? You didn’t tell me that!’ so I’ve been very open with all three children – Eleanor*

However, despite the mothers claiming to have been open and honest, most had withheld important details (Fig. 1) and were unsure how much detail to go into with their daughters:

*I’m not sure whether to go into the full depth of it, you know, that it could reduce their lives? – Alicia*

For the three daughters who had attended genetic counselling appointments, the mothers felt that this had improved their daughters’ understanding and knowledge of the carrier risk information and their reproductive options. Genetic counselling was highlighted as a way of overcoming the limitations of the mother’s medical knowledge, and it also tended to prompt further discussion and questions with the mother outside of the clinic:

[My first daughter] seemed to want to know more what would happen in circumstances if she was having a baby, what would happen - which obviously me and her Dad couldn’t answer.… So [the Genetic Counsellor] probably explained it more in detail to her medically more than me. Then she’d come back and was having a chat with us all and things about it – Fiona

### Barriers to Disclosure

The mothers unanimously felt that a lack of knowledge around the advances in genetic reproductive options had inhibited them from discussing this with their daughters. They were aware that reproductive techniques had progressed since they were last seen, but were unsure what the current options were for their daughters:

*Obviously I’ve tried to prepare them myself as much as I can, But then there’s other things like IVF and things like that, like new scientific things, and I’m not too clever on things like that – Belinda*

Two participants stated that they did not plan to discuss their daughters’ reproductive options ‘until she’s 16’ or ‘after she’s been tested, only if she’s positive’.

Concern for the future was one of the most significant inhibitors of disclosure and caused the mothers a great deal of anxiety. The reduced life expectancy associated with the condition proved extremely difficult for the mothers to accept, let alone to discuss with their daughters. They tended to completely avoid this topic as they were unsure how to best talk about it:

*I haven't actually asked to be honest because I don't want to upset her, whether she actually knows the full extent. I mean she knows he is going to get worse but I'm not sure beyond that… I’m not sure whether to go into the full depth of it, you know, that it could reduce their lives – Alicia*

Attempting to positively reframe this difficult information seemed to help the mothers cope, and two mothers repeatedly mentioned their faith in finding a cure. They had used this to try and lessen the impact of the information on their daughter, but in fact, this had led to them evading disclosure:

*She doesn’t know that it’s a terminal condition. I’ve kind of ‘prettied it up’ a bit. A lot of the time I’ve just said, ‘You know the way science is going forward and with everything that we do’ … I’ve said like, ‘Yes it’s a possibility, we can all die young.’ But I’ve pretty much said like that’s not going to happen – Claire*

The participants also struggled with concern around their daughters’ reproductive future and reported these conversations as some of the most difficult. They felt a great deal of fear and worry about their daughters having to make reproductive choices in the future:

*As it stands now I think what’s hardest is looking too far into the future and… well, thinking negatively really about her. I’m dreading her having a child, and I am very worried about the future for [my daughter] really – Alicia*

### Support and Information

There was a consensus from the mothers that there was a lack of professional advice and emotional support available to them and their daughters:

*We never really had any psychological support, or advice, or anything like that – Alicia*

Five of the participants were unsure about how to access support or advice about carrier risk disclosure, and none of them had sought or received advice from a genetic counsellor about communicating carrier risk information to their daughters. Only Fiona, whose daughter had recently been for a genetic counselling appointment, was aware that she could re-contact her genetic counsellor for advice and support but added that she ‘wouldn’t necessarily have known straight away who to speak to or who to go to for help’.

Participants suggested sending written information to mothers once they have had some time for their son’s diagnosis to settle in and can begin to think about the implications for their daughters, including a reminder of the basic facts, a summary of the reproductive options and advice for communicating with their daughters. They also stated a need for more psychological support for their daughters:

*Psychological support would have helped a lot. Just to deal with it with a little bit of support from elsewhere would have been nice… just somebody there, especially during the teenage years. – Alicia*

Many also recognised a need for additional psychological support themselves to overcome the carrier guilt and blame that they had been experiencing for years:

*Out of the whole thing no-one has ever tried to counsel me or offered anything to check how my wellbeing is about the fact that I’m a carrier – Claire*

Apart from Fiona (who was proactively contacted by the Genetics Department to offer a genetic counselling appointment to her daughters at age 16), the other participants thought the availability of genetics services needed to be made clearer so they would know that they could involve them proactively during the disclosure process, not just for carrier testing:

*Probably to involve the professionals more, and I might have let them do it more if they had been available, rather than me. But it wasn’t available so… They should have been available more to be involved, whereas it was left all for me to do… it was just a case of ‘well now it’s down to you to do it or not’, and there wasn’t really any support from them at all or any sort of guidance really - Deborah*

*Follow up families and check how they’re doing, and just giving them an option of being able to talk. To be able to just come in for a session to talk about how the whole thing makes them feel… or a leaflet or anything, I think just anything to let them know that there is help out there, and they’re not just on their own. – Claire*

Some participants thought it would be best for the Genetics Department to re-contact them when their daughters were ‘around 12 years old’; others thought ‘before 16 years old’.

The majority of participants bought up the ‘minimum age for carrier testing’ as an issue for carrier risk disclosure. They were under the impression that their daughters ‘could not have carrier testing until age 16’, so they felt it was unfair to tell their daughters about their risk at younger ages if they could not have genetic testing until they reached age 16, because it would cause them unnecessary worry and anxiety:

*Maybe it should be done now, sooner, so we can find out if they have, or if they haven’t. There’s no point putting them through all of this and talking to them over the years about it all and then when they have the test both of them are fine. It just seemed like a bit of an unnecessary… upset – Belinda*

They felt that it was important for families to be aware that earlier carrier testing is an option where appropriate. Although a couple of the mothers felt that the carrier testing should be done routinely at birth, the rest thought that the age limit for carrier testing should just be more flexible. One suggested that testing should be routinely offered at age of 14, as she felt that this was preferable to age 16 when they have the additional pressure of major school examinations and often more volatile emotions. One participant suggested that girls are often sexually active before age 16, so she felt it was important that they know if they are a carrier before then as she felt it would emphasise the importance of and encourage the use of contraception.

## Discussion

The participants’ experiences show that carrier risk disclosure is much more complex than simply giving their daughter a percentage chance of being a carrier. There is a process that involves the sharing of key pieces of information with their at-risk daughters at appropriate developmental stages. The information needed for the daughters to fully comprehend their carrier risks were the following: a general understanding about the condition, its genetics and inheritance; their chance of being a carrier; and their genetic testing and reproductive options. The timings of sharing this information varied between families but the order of information giving was consistent, not dissimilar from the three-staged model of disclosure of carrier risk information proposed by McConkie-Rosell et al. (2011).

Various studies have concluded that potential carriers were rarely aware of their carrier risk until age 16 (Fanos and Puck 2001; Metcalfe et al. 2011; Plumridge et al. 2010, 2011). Here, seven of the nine daughters were informed of their carrier risk at an average age of 10 years old, with conversations about the condition usually starting at an even younger age than previously reported by Plumridge et al. (2010).

Although it has previously been suggested that conversations between parents and children tended to focus on health implications rather than genetic risk (Plumridge et al. 2011), for the participants in this study, the converse was found. Mothers found it easier to discuss genetic risk and inheritance information than their daughters’ reproductive implications or the future of their sons’ health. This phenomenon may be specifically associated with DMD and BMD, possibly as a consequence of the progressive and life-limiting nature of the condition, or the X-linked pattern of inheritance.

All participants had endeavoured to be open and honest with their daughters, but they would often admit having withheld certain information. Avoidance also seemed to be a prominent issue around disclosure of the life-limiting nature of the condition. Mothers had recognised their daughters’ need for information but had often avoided cues or concealed information. They thought that genetic counsellors were better placed to share information on reproductive options, highlighting the benefits of genetic counsellor involvement with young people.

The general consensus in the literature is that disclosure of genetic information to children should be by the parents, but with the support and advice of health professionals (Forrest et al. 2008; McConkie-Rosell et al. 2009; Metcalfe et al. 2011; Plumridge et al. 2010; 2011). However, these participants’ experiences suggest that professional support is not being sought or effectively utilised. A striking observation was that it had not occurred to the participants to seek advice on carrier risk disclosure from health care professionals, and all but one were unaware that they could re-contact the Genetics Department for support, advice or information regarding this. Participants felt that a genetic counselling appointment should be proactively offered to their daughters before age 16, and that genetic counselling should be promoted as a source of help and guidance for parents during the disclosure process. They suggested that genetic counsellors could send out leaflets with information and advice for parents and daughters, reflecting Rowland and Metcalfe’s (2013) suggestion that a resource should be developed to include diagrams, disclosure techniques and examples of appropriate language to use. It also supports the ‘Recommendations for Health Care Professionals to support and aid disclosure of genetic information’ by Rowland and Metcalfe (2013) to use genetic counselling sessions with parents to explore, plan and prepare for disclosure to at-risk children, in a parent-practitioner model similar to that developed by Sullivan and McConkie-Rosell (2010). There was also evidence of inconsistency in how daughters were followed up by their genetics service and uncertainty about the age at which they could request carrier testing.

Helderman-van den Enden et al. (2013) propose testing at-risk females prenatally for DMD if foetal DNA is available or postnatally if not, which aligns with the opinions held by two of the mothers in this study. However, the majority of the participants favoured their daughters making an informed decision about carrier-testing themselves. Most felt that by age 14, their daughters would have the maturity and understanding necessary to make this decision and cope with the result. Guidance in the UK around consent in competent minors states that as long as a young person under age 16 is assessed to be ‘Gillick competent’, then they have the capacity to consent to genetic testing (Borry et al. 2005; Gillick v West Norfolk and Wisbech Area Health Authority 1985; Joint Committee on Medical Genetics 2011; British Society for Human Genetics 2010). Clearly, further study would be required to assess the outcomes of D/BMD carrier testing in young people under age 16.

### Study Limitations

This study was conducted in a single National Health Service (NHS) Genetics Centre in the North West of England, so it is important to note that other services may have different protocols for following up affected families and offering contact or information at certain time points. Therefore, the experiences and recommendations made by these participants may not be applicable to practice in other centres. This study involved a relatively homogenous sample of mothers in similar situations, who all believed they were coping well with the condition as a family and felt that they had ‘done the right thing’ in communicating with their daughters. Their experiences may differ from those of mothers who declined participation or did not feel that they were coping or communicating well with regard to their daughters’ carrier risks.

### Practice Implications

This study supports the case for genetic counsellors to build long-term relationships with families, rather than limiting themselves to a single, brief episode of contact. The participants made a number of suggestions for genetic counsellors (Table 3). Whilst these may not be wholly applicable to clinical practice in other genetics centres that follow different protocols in terms of maintaining long-term contact with affected families, it does provide some insight into the model of service they desire, which may be helpful to consider in the evaluation and long-term planning of services offered to families affected by D/BMD.

**Table 3:** Participants’ suggestions for genetic counsellors

Suggestion	Rationale
More proactive follow-up of families at key stages	• To allow genetic counsellors to aid the disclosure process • For mothers to feel more supported throughout • To improve the relationship between the genetic counsellor and the family • To make families more aware of the service
Sending written information and advice to the mothers and daughters when they reach specific ages	• To provide mothers with up to date information prior to disclosure to their daughters
Genetic counselling appointment to be proactively offered to the daughter (at around age 14)	• To aid the disclosure of reproductive information and options • To help create time for the daughter to process the information ahead of deciding about carrier testing
Genetic counselling appointment offered to the mothers	• To help address the mothers’ own feelings of carrier guilt and blame

### Research Recommendations

It would be beneficial to conduct a longitudinal follow-up study with these participants, especially those with younger daughters where the information had not yet been fully disclosed. It would also be important to consider the views of the children and young people at risk on when and how they would like to receive carrier risk information to understand their experience of carrier risk communication, compare and contrast their experiences and views about disclosure with that of their mothers and evaluate their support and information needs. As family dynamics clearly play an important role, this may best be achieved through family studies. The views of the affected sons and fathers could also be explored using a family approach. It would also be important to include the opinions of those not keen to participate in formal studies. D/BMD families from other genetics services and countries should be included in future studies, and it would be interesting to compare practices between different genetics centres to find out how other genetic counsellors are following up and supporting these families, and the effect this has on family communication.

## Conclusions

This exploratory study set out to address a gap in the research by investigating the experiences of mothers responsible for communicating DMD/BMD carrier risk information to their at-risk daughters. Carrier risk disclosure was found to be a process, with a pattern of information giving emerging in a particular order. All participants had struggled to a greater or lesser extent with some aspect of the process. The mothers gave the impression of full disclosure, claiming to have been completely open and honest with their daughters, but had often withheld important information leading to partial disclosure. There was a major barrier around discussion of the future, specifically discussing reproductive options and the life limitation of the condition. Genetic counsellors had a role in overcoming this barrier, by discussing reproductive options with the daughters at their clinic appointments.

The mothers felt that although they were the right people to inform their daughters about their chances of being a carrier, there should be much greater support and advice from genetic counsellors throughout the process. Their suggestions included sending written information and advice about genetic communication with at-risk daughter at certain stages, offering a genetic counselling appointment to the daughter at around age 14, and making the age limit for carrier testing more flexible. Further research would be required before any recommendations could be made for changes to genetic counsellor practice.

## Electronic supplementary material

ESM 1 (DOCX 26 kb)

## Footnotes

1 The term ‘family’ is used to describe parents and their children.

2 The research advisory group consisted of a consultant genetic counsellor, a professor of health care research and a lay mother of a daughter aged 16 years.
