# Supplementary material for: The Process of Disclosure: Mothers’ Experiences of Communicating X-Linked Carrier Risk Information to At-Risk Daughters
Source: J Genet Couns. 2018 Mar 19;27(5):1265–74. doi: 10.1007/s10897-018-0251-7 (PMC6132619; doi:10.1007/s10897-018-0251-7)
Supplement: Supplementary file 1 — (DOCX 26 kb) [file 10897_2018_251_MOESM1_ESM.docx]

*Journal of Genetic Counselling*

**The Process of Disclosure: Mothers’ Experiences of Communicating X-Linked Carrier Risk Information to At-Risk Daughters**

Amy Goldman ^1, 2^, Prof Alison Metcalfe ^3^ Dr Rhona MacLeod ^1, 2^

_1. Division of Evolution and Genomic Sciences, School of Biological Sciences, University of Manchester, Manchester, UK_

_2. Manchester Centre for Genomic Medicine, St Mary's Hospital, Manchester University NHS Foundation Trust, Manchester Academic Health Sciences Centre, Manchester, UK_

_3. Faculty of Health and Well-being, Sheffield Hallam University, Sheffield, UK_

*_Correspondence should be directed to:_* [*_amy.goldman@mft.nhs.uk_*](mailto:amy.goldman@mft.nhs.uk)

**Supplementary File**

**Semi-Structured Interview Guide**

Thank you for agreeing to take part– I know you must be busy so I really appreciate you taking the time to do this.

I’ve got some questions that I’d like to ask you, but if you think of anything at all please just add it in. I don’t want you to feel restricted by my questions, because really I’m just interested in hearing about your story and your ideas as a mum.

The first part is about how you learnt about Duchenne/Becker muscular dystrophy in your family. The second part’s more about talking to your daughter, whether that’s past present or future. The final part is asking about support and whether you have any idea’s or advice for us or for other parents.

**Your experience of learning about DMD/BMD**

1. I gather from your file that you’re a carrier of D/BMD, who is it in your family that is affected?
2. Can you tell me how old you were when you found out about the FH of Duchenne/Becker?
3. How did you first learn about this information?

(probe : Who told you? How were you told? Do you remember how you felt finding out?)

4) Who have you talked to in the family about Duchenne/Becker?

(probe: Who else have you told/spoken to about it in the family? Have you told anyone about your carrier status?)

5) How would you describe the communication in your family?

(probe: a) generally b) in relation to DMD/BMD? Is it something that is talked about quite a lot or something that’s kept more private?)

6) At what stage did you first find out that you might be a carrier of the condition yourself?

(probe: How did that come about? Were you seen in genetics at any stage? When did you first realise that it might mean that your daughter could also be a carrier? Do you remember how that felt when you found out?)

**Talking to daughter(s) about DMD/BMD**

1. Have you spoken to your daughter(s) yet about DMD/BMD at all?

(probe: What aspects of DMD/BMD have you talked about? What amount of detail did you go into? Did you have a similar conversation with both daughters? Did they react similarly or differently?)

**If yes:**

1. How did the conversation come about?

(probe: Had you discussed the issue with your partner beforehand? Had you planned for the conversation/your answers? How did the conversation with your daughter go? Did it bring up any unexpected emotions/questions? Has she come back to you with more questions since? Have there been further conversation as she’s got older?)

1. Have you talked specifically about the risk of your daughter being a carrier of the condition?

**If No:**

10) What sort of thoughts have you had about talking to your daughter about her carrier risk/the condition?

1. Have you decided yet when/if you plan to talk to your daughter about her carrier risk?

(probe: have you planned to talk to her at a specific age or stage? How do you imagine that the conversation with her will go? What makes you feel that would be a good time to broach the subject with her?)

12) How do/did you feel about the prospect of having this conversation with her?

(probe: do/did you feel prepared? Do/did you feel like you’d be able to answer all her questions?)

**Supporting you and your daughter**

13) Have you received any help/support in terms of talking to your daughter about her carrier risk? (probe: what sort of support? What about from her dad/other family members? Did you ever ask for or get any advice from professionals? Was there anything they said that was particularly helpful or unhelpful?)

14) Is there any type of support that would have been useful for talking to your daughter about all this?

(probe: can you think of any other type of support that might be useful to mum’s to help them feel comfortable talking to their daughter?)

e.g. meeting other families in similar situation/carrier mums

family/parent/child support groups

GC appt for you/daughter (at what age?)

age appropriate information leaflet for daughters

leaflet for parents about communicating with daughters

youtube video/mini documentary

website

1. What have you found (or anticipate finding) easiest about talking to your daughter about her carrier risk?
2. What have you found (or anticipate finding) hardest about talking to your daughter about her carrier risk?
3. Is there any advice you think you could give other parents in a similar situation about talking to their daughter?

**END**

18) Is there anything else you would like me to know?

If you or your daughter would like any advice or information in the future you’re always welcome to call us or book an appointment as you’re on our family register, so can come back in at any time.
